# Navigating Beauty Standards on Social Media: Impact of Appearance Activity on Adolescents’ Body Dissatisfaction

**DOI:** 10.1007/s10964-025-02159-y

**Published:** 2025-03-26

**Authors:** Nikol Kvardova, Hana Machackova, Chelly Maes, Laura Vandenbosch

**Affiliations:** 1 Interdisciplinary Research Team on Internet and Society, Faculty of Social Studies, Masaryk University, Jostova 10, Brno, Czech Republic; 2 Media Psychology Lab, KU Leuven, Leuven, Belgium

**Keywords:** Appearance activity, Social media, Tripartite influence model of body image, Body dissatisfaction, Adolescents

## Abstract

Social media activity focused on physical appearance can heighten body dissatisfaction in adolescents. However, the mechanisms behind this association remain insufficiently examined. This three-wave longitudinal study analyzed data from 2500 Czech adolescents (aged 11–16, *M* = 13.4, *SD* = 1.7, 50% girls) to examine whether the comparison with social media appearance ideals and the internalization of these ideals mediate the association between appearance activity on social media and body dissatisfaction. The bidirectional relationships and the differences between girls and boys were also explored. While significant between-person correlations were found over time, the within-person results showed that heightened appearance activity did not increase body dissatisfaction in subsequent waves. At the within-person level, social media-ideal internalization and appearance comparison did not mediate this connection. Although the heightened internalization of social media ideals predicted more appearance activity and appearance comparisons at the within-person level, these links were not consistent across waves. No significant differences were observed between adolescent girls and boys. This study indicates that appearance activity on social media do not necessarily reinforce adolescents’ body dissatisfaction six months later, providing insights for both research and policy.

## Introduction

Adolescents routinely engage with appearance activity on social media (Schreurs & Vandenbosch, 2022), including browsing and posting images of idealized appearances, which are often promoted by “likes” and comments (Zimmer-Gembeck et al., 2021). These activities have been linked to increased body dissatisfaction among adolescents (Holland & Tiggemann, 2016). However, the mechanisms underlying this relationship remain scarcely studied. Much of the existing research relies on cross-sectional data, fails to focus on specific appearance-related activities (Saiphoo & Vahedi, 2019), or, in the case of limited longitudinal studies, fails to distinguish between within-person and between-person effects (de Valle et al., 2021). Guided by the Tripartite Influence Model of body image (Thompson et al., 1999), this study employed three-wave longitudinal data from adolescent girls and boys to investigate whether internalization and comparison with social media ideals mediate the relationship between appearance activity on social media and body dissatisfaction. Additionally, the study examined bidirectional associations and explored whether there are differences between girls and boys in these dynamics.

### Role of Appearance Activity on Social Media in Body Dissatisfaction Among Adolescents

Adolescence is a critical period for the development of body dissatisfaction (McLean et al., 2022). During early adolescence, pubertal changes, marked by weight gain and shifts in body shape, typically move girls away from the thin-ideal while boys typically move closer to the muscular-ideal (Ricciardelli & Yager, 2016). Other developmental changes include increased abstract thinking and perspective-taking, the growing salience of peer relationships and social status, and an orientation toward romantic dating. These all stimulate the focus on the body and the importance of being accepted by peers based on physical appearance (Choukas-Bradley et al., 2022). Identity development also encompasses the formation of attitudes related to physical self-concept and body image (Salomon & Brown, 2019).

The combination of these factors makes early and middle adolescents particularly vulnerable to experiencing body dissatisfaction and to the influence of social media. Social media use increases rapidly from the age of 12 onward (Smahel et al., 2020), while its novelty at this developmental stage may intensify the impacts on adolescents (Vandenbosch et al., 2025). Adolescents use social media intensely to connect with friends, share and search for information, pass time, escape their daily lives, and seek appearance feedback (Jarman et al., 2021a, ). For appearance purposes, visually-oriented social media, like Instagram, TikTok, and Snapchat, are particularly prominent (Vandenbosch et al., 2022), with adolescents engaging with them daily (Pew Research Center, 2023). These platforms display so-called “appearance content”, which contains imagery and text focused on physical appearance, including posts and stories with “selfies”, pictures, and videos of others’ bodies and facial appearances in order to promote appearance ideals, make-up style, clothing style (Schreurs & Vandenbosch, 2022), fitness, diet, and excessive exercise (Tiggemann & Zaccardo, 2018).

Considering the developmental tasks of adolescence, appearance activity can be understood in a developmental context, because adolescents often engage with such activity for self-presentation, identity exploration, social status, and the peer approval of appearance (Choukas-Bradley et al., 2022). Compared to traditional media, social media feature user-generated and self-relevant content from close targets, like friends and peers (Fardouly & Vartanian, 2016). Furthermore, a large portion of appearance content is highly idealized because users selectively post their best images and apply filters and modification techniques (Fardouly et al., 2020). That being said, social media is full of attractive appearances and idealized thin and muscular bodies. Adolescents typically strive to look attractive by comparing themselves to attractive peers and celebrities on social media and posting idealized versions of themselves to seek positive feedback (Rodgers & Rousseau, 2022). As appearance becomes a major focus for adolescents, with peer opinions shaping what adolescents perceive as attractive (Markey, 2010), the appearance ideals disseminated on social media guide their appraisal of attractiveness and shape their body image. As a result, visually-oriented social media are believed to intensify the focus on idealized appearances of oneself and others in adolescence, potentially reinforcing the internalization of attractive ideals and heightening body dissatisfaction (Choukas-Bradley et al., 2022).

A substantive body of research has shown that exposure to idealized thin and muscular social media images intensifies body dissatisfaction among adolescent girls and boys (Saiphoo & Vahedi, 2019). However, due to the interactive features of social media, adolescents can engage with appearance content in various ways. These activities encompass taking, editing, and (re)posting “selfies” (McLean et al., 2019), photos of others, and appearance-related written posts; and “liking” others’ appearance content and writing comments (Schreurs & Vandenbosch, 2022). Additionally, the activities involve browsing appearance content, including “likes” and comments, from other users (Fatt & Fardouly, 2023). Research has shown that it is not the general time spent on social media, but the engagement in appearance activities that significantly shapes body image (Cohen et al., 2017; Saiphoo & Vahedi, 2019).

Despite the growing diversity of visually-driven content on social media, like the emergence of the body positivity movement (Cohen et al., 2021), idealized portrayals persist. This study focused on appearance activity centered on body shape and body ideals, which, on social media, are mostly in the form of attractive thin and muscular bodies (Raiter et al., 2023). Mirroring the evidence on non-interactive exposure (Saiphoo & Vahedi, 2019), correlational studies have indicated that appearance activity on social media may contribute to elevated body dissatisfaction in adolescents. Browsing others’ photos on Instagram was associated with decreased body esteem among adolescent girls aged 12–16 (Chang et al., 2019). Engaging with photo-related activities on Facebook, like updating profile pictures or viewing friends’ photos of themselves, was linked to increased body dissatisfaction in adolescent girls aged 12–17 (Scully et al., 2023). Among adolescents aged 12–19, viewing selfies on social media was associated with increased facial dissatisfaction (Wang et al., 2019).

### Mediating Roles of Social Media-Ideal Internalization and Appearance Comparison

The Tripartite Influence Model of body image (Thompson et al., 1999) posits that society continuously emphasizes the importance of looking attractive and having an ideal thin or muscular body. This pressure is propagated via *media*, *peers*, and *parents*, prompting individuals to internalize these ideals and compare themselves to them (Thompson et al., 1999). While appearance comparison refers to evaluating one’s own body against idealized standards, internalization involves adopting these standards as personal goals and striving to attain a body that aligns with them (Karazsia et al., 2013). Achieving these ideals proves challenging, because most experience a gap between the idealized and the actual body, which amplifies body image concerns (Frederick & Reynolds, 2022).

Although originally developed for traditional media, the Tripartite Influence Model (Thompson et al., 1999) also extends to appearance activity on social media and body dissatisfaction. Nowadays, social media serve as a primary source for body ideals. Compared to traditional media, social media allow interactions and active engagement with ideal bodies with “likes”, comments, posts, and shares (Fatt & Fardouly, 2023). Media and peers are closely intertwined in today’s social media landscape, where the appearance content viewed, shared, and posted can be endorsed or disapproved by others. That said, both “passive” (e.g., browsing) and “active” (e.g., posting, liking) engagement occur there. According to Rodgers (2016), both browsing idealized content and actively creating idealized self-presentations can contribute to body dissatisfaction, with the internalization of and comparison to appearance ideals playing a significant mediating role (Rodgers et al., 2020). While exposure to idealized bodies reinforces internalization and appearance comparisons, as suggested by the original model (Thompson et al., 1999), posting idealized versions of oneself (or others) on social media can emphasize the gap between the ideal and actual appearance, further contributing to body dissatisfaction (Rodgers, 2016). This aligns with the Self-Effects Framework, which demonstrates how posting behaviors on social media reflects back on emotions, cognitions, and attitudes (Valkenburg, 2017).

So far, there is empirical support for the Tripartite Influence Model (Thompson et al., 1999) processes for appearance-related social media. A recent meta-analysis showed appearance comparison and internalization to mediate the link between exposure to idealized images on social media and body image concerns within experimental and longitudinal research, although the evidence is not yet extensive and fully consistent (de Valle et al., 2021). Specifically for the topic of appearance activity on social media and body dissatisfaction in adolescents, most results come from cross-sectional studies, where appearance comparisons mediated the association between browsing and editing selfies and heightened body image concerns among adolescent girls aged 12–16 (Chang et al., 2019). The internalization of the importance of looking attractive mediated the association between selfie viewing and facial dissatisfaction in adolescents aged 12–19 (Wang et al., 2019). Among adolescents aged 12–17, posting selfies and viewing how others commented correlated with decreased body satisfaction, mediated by appearance comparison and the internalization of general attractiveness, which is the internalization of the importance of looking attractive (Scully et al., 2023). Utilizing longitudinal data, Jarman et al. (2024) supported the Tripartite Influence Model (Thompson et al., 1999) by finding that thin-ideal internalization and social media comparisons mediated the association between using visually-oriented social media and engagement in photo-based activities on decreased body satisfaction six months later in adolescents aged 11–16.

Despite this line of work, the mediating variables of internalization and appearance comparison can work differently in the context of social media compared to traditional media. The current study focuses specifically on the internalization of attractive bodies and appearance comparisons on social media, with a particular emphasis on the sequence in which these processes unfold. This responds to calls from previous body image scholars to dedicate attention to the chain of events and the order in which body image processes emerge (Vangeel et al., 2018). Initially, the Tripartite Influence Model (Thompson et al., 1999) proposed that appearance comparisons and the internalization of beauty ideals operate simultaneously in conveying media and peer appearance pressures on body dissatisfaction (Thompson et al., 1999). While ample research demonstrates this for traditional media (Keery, Van Den Berg, et al., 2004; Lovering et al., 2018; Rodgers et al., 2011), a sequential order in these mediating variables can occur for social media. As such, the internalization of social media ideals can drive increased appearance comparisons and subsequently heighten body dissatisfaction. Rodgers et al. (2015) advocated for this serial association, drawing from the Social Comparison Theory (Festinger, 1954), which suggests that comparisons may arise from areas of interest and self-improvement motives. The stronger internalization of media-ideal standards prompts more frequent appearance comparisons because achieving the ideal body becomes more relevant and important. A longitudinal study supported this view: heightened media-ideal internalization predicted increased appearance comparisons among early adolescent girls, which in turn predicted higher levels of body dissatisfaction over time (Rodgers et al., 2015). The reverse pathway, where appearance comparison intensifies internalization, may also be at play, but it has not yet been supported. Still, it is likely that those who compare themselves to attractive media images are more inclined to internalize their desirability (Rodgers et al., 2015).

A couple of cross-sectional studies suggested a serial order for internalization and appearance comparison in mediating the link between appearance activity on social media and body dissatisfaction. Specifically, internalization mediated the connection between appearance comparison and body dissatisfaction (Lee & Lee, 2021; Piccoli et al., 2022; Scully et al., 2023), and comparison mediated the relationship between internalization and body dissatisfaction (Jarman et al., 2021b; Lee & Lee, 2021). However, cross-sectional data is limited in its ability to distinguish between the parallel and serial orders in the mediating processes of social media-ideal internalization and appearance comparison. There are limited longitudinal studies that investigated the association between social media use and adolescent body image over time (Course-Choi & Hammond, 2021). Even fewer longitudinal studies specifically focus on media-ideal internalization and appearance comparison as mediating processes.

Furthermore, available studies did not differentiate between within-person and between-person effects. This distinction is particularly relevant to the topic of appearance-focused social media and body dissatisfaction in adolescents (Schreurs & Vandenbosch, 2022). The importance of differentiating within-person and between-person effects has recently gained attention in the field of psychology (Borsboom & Haslbeck, 2024). Echoing this debate, it has been shown that the relationship between social media use and well-being exhibits distinct patterns at the between-person and within-person levels (Stavrova & Denissen, 2021). Yet, most longitudinal research on engaging with appearance-related social media and body image has thus far conflated these two distinct types of evidence, as shown by meta-analyses (de Valle et al., 2021; Saiphoo & Vahedi, 2019). As a result, these prior studies have shed light on whether individuals with higher engagement with appearance-related social media (compared to others) experience heightened internalization, comparisons, and body dissatisfaction over time (Jarman et al., 2024). However, they cannot enlighten when internalization and comparison emerge and whether changes in appearance activity lead to subsequent changes in body dissatisfaction. Building on this, the present study aimed to disentangle the longitudinal associations between appearance activity on social media, internalization, comparisons with social media ideals, and body dissatisfaction in adolescents, while distinguishing these at the within-person and between-person levels.

### Exploration of Bidirectionality and Differences Among Girls and Boys

An additional gap in the current literature is the lack of insight into the bidirectional links between appearance activity on social media, appearance comparison, internalization, and body dissatisfaction. While appearance activity can contribute to later body dissatisfaction (de Valle et al., 2021), body dissatisfaction, along with internalization and appearance comparison, may also drive adolescents to engage more frequently in appearance activity. This assumption is in line with the uses and gratification perspective applied to the media-body image context (Rodgers, 2016). Adolescents with higher levels of internalization, comparison, and body dissatisfaction may search for and interact with idealized content on social media to satisfy their inclination toward appearance ideals. According to the meta-analysis (de Valle et al., 2021), a couple of longitudinal studies have highlighted that body image motivates specific social media behaviors, like those with poorer body image add more friends on social media (Tiggemann & Slater, 2017) and monitor attractive peers (Vandenbosch & Eggermont, 2016), though these studies did not distinguish between within-person and between-person effects. Hence, it is warranted to disentangle whether, at the within-person level, changes in body dissatisfaction, internalization, or comparison can drive subsequent changes in appearance activity.

In addition, this study looked into potential differences between girls and boys. Girls and boys are typically targeted with different ideals: girls with thinness, boys with muscularity (Murnen & Don, 2012). Girls also face higher objectification and invest more in appearance (Choukas-Bradley et al., 2022). Studies suggest that girls engage more intensely with appearance-focused content on social media (Paddock & Bell, 2021). These inclinations may lead to greater involvement in appearance activity on social media and higher susceptibility to its impact on social media-ideal internalization, appearance comparison, and subsequent body dissatisfaction in girls compared to boys. However, similarly strong connections between appearance-focused social media use and body satisfaction have been discovered among girls and boys aged 11–17, mediated by internalization and appearance comparison (Jarman et al., 2021a, 2024). The equivalence of social media effects on body image between girls and boys has further been documented in the recent meta-analysis (Saiphoo & Vahedi, 2019). Although research has shown that boys and girls use social media differently, such as girls using more photo-based platforms and taking and editing more selfies than boys, research generally hints that the links between appearance-based social media use and body image are similar in these groups (Vandenbosch et al., 2022). However, further exploration of these differences in the engagement with appearance activity on social media and body dissatisfaction is needed, especially in regards to the roles of the mediating factors of internalization and appearance comparison with social media ideals.

### Confounding Variables

The links between appearance activity on social media, internalization, comparison with social media ideals, and body dissatisfaction can be influenced by additional variables, namely time spent on social media, depressive moods, BMI, sex, and age. While the present study examined appearance activity on social media that was centered on idealized content, using social media encompasses various body image-related experiences, such as receiving positive comments that encourage body acceptance and engaging with body positivity content that challenges beauty ideals (Rodgers et al., 2021). Engaging with these contradictory content types can intertwine in social media use among adolescents (Stevens & Griffiths, 2020). Furthermore, depressive moods and BMI can contribute to body dissatisfaction because adolescents who experience these moods and have larger bodies typically report higher levels of body dissatisfaction (Rodgers et al., 2014). Lastly, while girls and boys can have different appearance-related experiences on social media and body dissatisfaction (Paddock & Bell, 2021), age can also play a significant role. Adolescents encounter various developmental challenges during early to middle adolescence (Rodgers & Rousseau, 2022) and may respond differently to appearance activity on social media with respect to their body dissatisfaction.

## Current Study

While appearance activity on social media has been cross-sectionally linked to increased body dissatisfaction among adolescents, there is limited insight into the processes that underlie this relationship over time. Building on the Tripartite Influence Model of body image (Thompson et al., 1999), the present study examined whether the relationship between appearance activity on social media and body dissatisfaction is mediated by social media-ideal internalization (Hypothesis 1) and appearance comparison on social media (Hypothesis 2) in adolescent girls and boys. Furthermore, the study looked into the bidirectional associations between appearance social media activity, the internalization of social media ideals, appearance comparison, and body dissatisfaction, and how these vary between adolescent girls and boys. This study focused on these questions using a random-intercept cross-lagged panel model to separate within-person and between-person dynamics over time. Although the key questions were examined at the within-person level, the between-person associations were also explored, because they can provide valuable insights for both research and policy. The models additionally accounted for time spent on social media, depressive moods, BMI, sex, and age.

## Methods

### Participants

This study uses data from three waves that were carried out six months apart (W_1_: June 2021, W_2_: December 2021, W_3_: June 2022). In the first wave, 2500 adolescents aged 11–16 (*M* = 13.4, *SD* = 1.7, 50% girls) took part. In the subsequent waves, 1654 (33.8% attrition from W_1_; *M*_*age*_ = 13.7, *SD*_*age*_ = 1.8, 48% girls) in the second and 1102 (33.4% attrition from W_2;_ *M*_*age*_ = 14.2, *SD*_*age*_ = 1.8, 48% girls) in the third wave completed the questionnaire. Sex and age were evenly distributed in the first wave. The socioeconomic status of households, indicated by the highest education level of the household head and residence and categorized by NUTS3 region and municipality size, were representative of Czech households with children.

### Procedure

The data were collected as part of the project *Modeling the future: Understanding the impact of technology on adolescents’ well-being*, which received approval from the Research Ethics Committee of Masaryk University (ref. number: EKV-2018-068). The data collection adhered to the ethical guidelines set by ESOMAR and SIMAR. The data collection was conducted via online questionnaire and managed by the STEM/MARK agency. The data were collected from both adolescents and their parents/caregivers, one parent and one adolescent per household, though the present study utilized data from adolescents only. The agency contacted the potential respondents from their online panel via e-mail. Initially, 17,502 adult panel members were invited to participate, with the invitation asking them and their one adolescent child to participate. The email invitations outlined the study, eligibility criteria, the questionnaire procedure, and included a link to a PDF version of the adolescent questionnaire, allowing parents to preview the questions before deciding to participate. Active informed consent from adolescents and one parent (or caregiver) was mandatory. For subsequent waves, parents were contacted via email. They were asked control questions (e.g., previous wave participation) and instructed to ensure that the same child completed the questionnaire as in the prior wave.

Upon starting the online questionnaire, parents confirmed their child’s eligibility, which required them to be an internet user. For the first wave, parents also confirmed quotas: adolescent’s sex and age, household’s socioeconomic status, place of residence, and municipality size. After that, they granted permission for the adolescent to participate and were instructed to provide the device, whether a laptop/desktop, smartphone, or tablet, with the online questionnaire to the adolescent. Parents were urged to ensure the adolescents’ privacy during the questionnaire process. Adolescents had the option to refrain from answering any question and they could exit the questionnaire at any time. After completing the questionnaire, adolescents were debriefed and informed that clicking “continue” would finalize their answers and prevent parental viewing. The median duration for completing the questionnaire was 30 minutes. The variables used in the study encompassed media use and various aspects of adolescents’ well-being. Attrition in subsequent waves was associated with factors such as non-response to invitations, non-completion of the survey (the agency delivered only “valid” questionnaires, which were defined as those with less than 10% missing data resulting from selecting “I don’t want to respond”), and elimination during data quality control and processing. For eligibility reasons, adolescents and parents were also asked whether they had completed the questionnaire in the previous wave. In the second wave, none responded “No”; 95% of parents/caregivers answered “Yes” and 5% were not sure, while 91% adolescents stated “Yes” and 9% were not sure. In the third wave, 97% parents stated “Yes” and 3% were not sure; among adolescents, 94% said “Yes” and 6% were not sure. No participants were excluded based on their responses to these questions. The questionnaire items were translated from English to Czech collaboratively by the first and the second authors of this study. The first author drafted the initial translation. In the next phase, both authors worked together to refine the translation, ensuring optimal comprehensibility while maintaining the original meaning. The study was preregistered at the Open Science Framework (OSF) platform (https://osf.io/6acxg).

Across the waves, the proportion of participants who answered “I don’t want to respond” reached 4% for body dissatisfaction items, 1.5% for social media-ideal internalization items, 1.4% for appearance comparison items, and 1.4% for appearance activity items. In addition, to find out if the attrition rates depended on the key variables (i.e., appearance activity on social media, body dissatisfaction, social media-ideal internalization, appearance comparison on social media, depressive mood, BMI, time spent on social media, age, sex), two logistic regression models were conducted. The first one predicted attrition from W1 to W2; the second predicted attrition from W2 to W3. The attrition from W1 to W2 did not depend on any key variable. The results showed that attrition from W2 to W3 depended significantly on appearance activity (β = −0.21, *OR* = 0.80, *p* = 0.005) and with a marginal *p*-value on appearance comparison on social media (β = 0.18, *OR* = 1.18, *p* = 0.045), which indicates that adolescents who were less engaged with appearance activity and reported more appearance comparison were more likely to be included in the sample.

### Measures

#### Appearance activity on social media

Appearance activity was assessed with the Appearance Activity Subscale of the Social Media Appearance Preoccupation Scale (Zimmer-Gembeck et al., 2021). The original scale consists of six items. Three concerned exposure to posting, sharing, commenting, and the liking of appearance social media content, and the other three asked about doing these actively. Slight adaptations were made. First, the instructions were changed from “friends” to “people you follow on social media”, since the content from more distant users can also influence body dissatisfaction. Second, in the “When on social media I/my friends post, comment on, share or like content about what and when to eat” item, “for having a nice body” was added, since the original item could include dieting choices that were not for the purpose of body-change. Participants were asked, “On social media, you can sometimes see content (photos, videos, texts) focused on body appearance. Would you say that the people you follow on social media post, share, comment, or like… content about what they would like their bodies to look like, … content about how to eat to have a nice body, (or) … content about various things that can be done to be lean and/or muscular”. “And would you say that you yourself, on social media, post, share, comment, or like… content about what you would like your body to look like, … content about how to eat to have a nice body, (or) … content about various things that can be done to be lean and/or muscular”. The response scale ranged from (1) Strongly disagree to (5) Strongly agree. A higher score reflects a higher engagement with appearance activity on social media. Since the appearance activity variable was not assumed to be represented by a reflective latent variable, its internal consistency or invariance were not assessed (see Gruijters et al., 2021).

#### Body dissatisfaction

Body dissatisfaction was assessed with the modified Body Dissatisfaction Subscale of the Eating Disorder Inventory-3 (Garner, 2004). The five items that inquired about satisfaction with the thighs, buttocks, abdomen, hips, and overall body shape were used. The omitted items from the original scale were the reversely coded ones that asked about these body areas in a reverse manner. Participants were asked, “Each of us perceives ourselves differently. Some people like how they look, and some are not entirely satisfied with it. Are you satisfied or dissatisfied with the appearance of … your figure, … your thighs, … your abdomen, … your hips, (and) … your buttocks”. The response scale ranged from (1) Very dissatisfied to (5) Very satisfied. The items were reversely coded to obtain a score of body dissatisfaction. A higher score reflects a higher body dissatisfaction. Internal consistency was sufficiently high across waves (ω_W1_ = 0.92, ω_W2_ = 0.93, ω_W3_ = 0.94).

#### Social media-ideal internalization

Social media-ideal internalization was measured with the adapted Sociocultural Internalization of Appearance Questionnaire – Adolescents (Keery, Shroff, et al., 2004). The scale for measuring the internalization of traditional media ideals was adapted to social media. Participants were asked, “On social media, you can also sometimes see photos or videos of girls [in the female version] / boys [in the male version] you think have a great figure. When you see these [girls / boys] on social media, how much do you agree or disagree with the following: I want my body to look like theirs; I would like to change something in my appearance; I would like to lose or gain weight; I would like to change the way my body looks; I would like to look just like them”. The response scale ranged from (1) Strongly disagree to (5) Strongly agree. A higher score reflects a higher social media-ideal internalization. Internal consistency was sufficiently high across waves (ω_W1_ = 0.92, ω_W2_ = 0.94, ω_W3_ = 0.94).

#### Appearance comparison on social media

Appearance comparison on social media was assessed with the adapted Physical Appearance Comparison Scale-3 (Schaefer & Thompson, 2018). The original scale consists of questions about comparing the overall appearance, body size, body shape, and weight in various offline contexts (e.g., school, friends, party). In this study, comparisons made on social media were assessed with “On social media, you can also sometimes see photos or videos of girls [in the female version] / boys [in the male version] you think have a great figure. When you see girls [in the female version] / boys [in the male version] with such a great figure on social media, how often do you do the following: I compare my appearance with them; I compare the size of some parts of my body (e.g., abdomen, thighs) with them; I compare the shape of my figure with them; I compare my weight with them”. The response scale ranged from (1) Never to (5) Very often. A higher score reflects a higher appearance comparison. The internal consistency was sufficiently high across waves (ω_W1_ = 0.95, ω_W2_ = 0.95, ω_W3_ = 0.94).

#### Time spent on social media

Time spent on social media was measured by asking “In the last 6 months, how often have you used a social networking site where you have a profile (e.g., Instagram, Facebook, TikTok)? We don’t mean messaging-only apps like WhatsApp or Messenger.” The response scale was 1 (Never), 2 (At most a few times), 3 (Several times a month), 4 (Several times a week), 5 (Every day), 6 (Several times a day). A higher score stood for higher frequency of use.

#### Depressive moods

The propensity for depressive mood was measured with four items adapted from Kandel & Davies (1982). Adolescents were asked how often they experienced selected worries in the past months (i.e., “I felt unhappy or sad”, “The future seemed hopeless to me”, “I felt tense or restless”, or “I was very worried”). The response scale ranged from 1 (Never) to 6 (Very often). A higher score indicated a higher propensity for depressive mood. The internal consistency was sufficiently high across waves (ω_W1_ = 0.85, ω_W2_ = 0.88, ω_W3_ = 0.88).

#### Body mass index (BMI)

The Body Mass Index (BMI) was computed from reported height (“What is your height? State in cm”) and weight (“What is your weight? State in kg”).

#### Sex

Sex was asked with “You are:” with the options to answer “A girl” or “A boy”.

#### Age

Age was inquired with “How old are you?” with an open-ended response format.

### Data Analysis

The descriptive statistics were computed, alongside the scales’ internal consistencies (except for the appearance activity scale, which was not expected to be underlaid by a latent variable; Gruijters et al., 2021), and the zero-order correlations across the measurement points. To assure meaningful comparisons across time and sex groups, the measurement invariance was tested for the body dissatisfaction, social media-ideal internalization, appearance comparison on social media, and depressive mood scales. The measurement invariance was not tested for appearance activity because it was not assumed to have an underlying latent variable. The configural (i.e., fixed factor structure), metric (i.e., fixed factor loadings), and scalar (i.e., fixed intercepts) invariance levels were examined, following good/acceptable criteria of RMSEA ≤ 0.06/0.08, SRMR ≤ 0.08, TLI/CFI ≥ 0.95/0.90 (Hu & Bentler, 1999) for the configural model; for the metric and scalar models, acceptable differences from the less-restricted model were CFI ≤ 0.01, ΔRMSEA ≤ 0.015, and ΔSRMR ≤ 0.030, and ΔCFI ≤ 0.01, ΔRMSEA ≤ 0.015, and ΔSRMR ≤ 0.015, respectively (Chen, 2007).

Finally, the random intercept cross-lagged panel model (RI-CLPM; Hamaker et al., 2015) was set up. RI-CLPM separates the trait-like stable between-person average expected scores, represented by random intercepts, and the within-person fluctuations from these expected scores. This enables the exploration of the within-person dynamics while accounting for the stable between-person components of the studied variables over time (Hamaker et al., 2015). Here, RI-CLPM included appearance activity on social media, social media-ideal internalization, appearance comparison on social media, and body dissatisfaction, each measured at three time points. Depressive mood, BMI, time spent on social media, sex, and age, each measured in the first wave, were included as covariates. Both between-person and the within-person relationships between appearance activity, internalization, appearance comparison, and body dissatisfaction were examined. The robust maximum likelihood estimation (MLR) was used, except for the mediation analysis, since the bootstrapping procedure could not be run with the robust estimator. Full information maximum likelihood (FIML) was used to handle missing data. This approach enables the use of all available data despite missingness resulting from attrition or non-response. The model fit was evaluated following the criteria TLI ≥ 0.90, RMSEA ≤ 0.08, SRMR ≤ 0.10 (Kline, 2012).

First, the variance and correlations of random intercepts were inspected, which were interpreted as between-person associations for appearance activity on social media, social media-ideal internalization, appearance comparison, and body dissatisfaction. Second, the within-person associations in these variables were inspected, including the reciprocal ones. Third, the mediated effects of social media-ideal internalization and appearance comparison on social media were examined, mediating the relationship between appearance activity on social media and body dissatisfaction. The mediated effects were assessed with the product method (Cheng et al., 2021), which tests the statistical significance of the multiplied products of the predictor-mediator path (i.e., from appearance activity to internalization or appearance comparison) and the mediator-outcome path (i.e., from internalization or appearance comparison to body dissatisfaction). The statistical significance was examined with the bootstrapping procedure. The mediated effects were considered significant at the 0.05 level when the 95% confidence intervals excluded zero. Fourth, differences between girls and boys were explored with the multi-group RI-CLPM by testing the differences between the model with the within-person cross-lagged pathways constrained to be equal across girls and boys, and the unconstrained model with freely estimated pathways, following the procedure of Mulder and Hamaker (2021).

In addition, several alternative models as part of a sensitivity analysis were run to ensure the robustness of the findings. Specifically, the associations between appearance activity on social media, social media-ideal internalization, appearance comparison on social media, and body dissatisfaction were examined by running the model while (a) including exposure (i.e., seeing others post, share, comment, or like appearance content on social media) and interactive appearance activities (i.e., one’s own posting, sharing, commenting, or liking of such content) separately, and (b) not incorporating covariates (i.e., BMI, depression, sex, age, time spent on social media).

### Deviations from Preregistration

Contrary to the preregistered plan, the residual covariance between the two indicators of the social media-ideal internalization scale (i.e., “I want my body to look like theirs”, “I would like to look just like them”) was allowed to achieve better model fit, since this was likely a result of the nearly identical wording. Due to excessive complexity and the RI-CLPM’s convergence issues, the covariate variables were not included as time-variant with their own random intercepts but as time-invariant and measured in the first wave only.

## Results

### Preliminary Results

The means, standard deviations, and zero-order correlations are displayed in Table 1. Overall, reported body dissatisfaction was low. In the first wave, 11% of adolescents indicated being rather or very dissatisfied with their bodies. In the second and third waves, it was 8% and 11%, respectively. Across waves, appearance activity correlated positively and significantly with social media-ideal internalization (*r* = 0.23–0.48) and appearance comparison on social media (*r* = 0.30–0.54). Similarly, body dissatisfaction showed positive correlations with internalization (*r* = 0.29–0.50), appearance comparison (*r* = 0.29–0.38), and appearance activity on social media, though the correlation with appearance activity was rather week (*r* =0.02–0.12). As reported below, a similar pattern was observed for between-person correlations within the RI-CLPM.

**Table 1:** Zero-order correlations

	*M*	*SD*	1	2	3	4	5	6	7	8	9	10	11	12	13	14	15	16	17
Activity (W1)	2.71	0.93																	
Activity (W2)	2.68	0.92	0.47*																
Activity (W3)	2.70	0.92	0.43*	0.47*															
Internalization (W1)	2.92	1.09	0.39*	0.30*	0.33*														
Internalization (W2)	2.85	1.1	0.28*	0.51*	0.37*	0.58*													
Internalization (W3)	2.91	1.07	0.23*	0.28*	0.48*	0.54*	0.63*												
Comparison (W1)	2.31	1.09	0.45*	0.36*	0.37*	0.66*	0.49*	0.47*											
Comparison (W2)	2.32	1.07	0.36*	0.54*	0.41*	0.51*	0.70*	0.52*	0.60*										
Comparison (W3)	2.40	1.04	0.30*	0.35*	0.50*	0.50*	0.55*	0.72*	0.59*	0.64*									
BD (W1)	2.55	0.97	0.02	0.05*	0.09*	0.49*	0.38*	0.38*	0.34*	0.30*	0.32*								
BD (W2)	2.46	0.93	0.04	0.11*	0.12*	0.41*	0.50*	0.43*	0.29*	0.37*	0.35*	0.61*							
BD (W3)	2.48	0.96	0.01	0.09*	0.10*	0.39*	0.44*	0.51*	0.29*	0.33*	0.38*	0.60*	0.65*						
Depression (W1)	2.73	0.83	0.17*	0.22*	0.19*	0.35*	0.30*	0.30*	0.36*	0.32*	0.32*	0.30*	0.28*	0.26*					
BMI (W1)	20.58	4.5	0.02	−0.05	0.03	0.19*	0.14*	0.17*	0.13*	0.10*	0.10*	0.32*	0.25*	0.26*	0.03				
Social media (W1)	4.22	1.75	0.16*	0.10*	0.12*	0.23*	0.22*	0.17*	0.23*	0.23*	0.19*	0.09*	0.09*	0.07*	0.14*	0.09*			
Age (W1)	13.7	1.8	0.10*	0.06*	0.06	0.09*	0.06*	0.05	0.14*	0.09*	0.06	0.03	0.04	0.05	0.16*	0.19*	0.28*		
Sex (W1)			−0.12*	−0.14*	−0.13*	−0.15*	−0.15*	−0.12*	−0.23*	−0.22*	−0.20*	−0.11*	−0.10*	−0.12*	−0.14*	0.09*	−0.14*	0.02	

Sex was coded as 1 = Girl, 2 = Boy

*Activity* Appearance activity on social media, *Internalization* Social media-ideal internalization, *Comparison* Appearance comparison on social media, *BD* Body dissatisfaction, *Depression* Depressive mood, *Social media* Time spent on social media

*p < 0.050

Overall, the scales showed satisfactory results for measurement invariance, both longitudinally and between the girls and boys groups. In terms of longitudinal invariance, scalar models with constrained intercepts showed satisfactory model fit for body dissatisfaction (χ^2^(93) = 370.596, TLI = 0.975, CFI = 0.978, RMSEA = 0.035 (90%CI: 0.031, 0.038), SRMR = 0.078); social media-ideal internalization (χ^2^(90) = 334.565, TLI = 0.981, CFI = 0.984, RMSEA = 0.033 (90%CI: 0.029, 0.037), SRMR = 0.031); and appearance comparison on social media (χ^2^(56) = 83.270, TLI = 0.997, CFI = 0.998, RMSEA = 0.014 (90%CI: 0.007, 0.020), SRMR = 0.036). Similarly, scalar invariance was supported across girls and boys for body dissatisfaction (χ^2^(194) = 580.948, TLI = 0.967, CFI = 0.970, RMSEA = 0.040 (90%CI: 0.036, 0.044), SRMR = 0.060); social media-ideal internalization (χ^2^(189) = 1589.565, TLI = 0.899, CFI = 0.909, RMSEA = 0.077 (90%CI: 0.074, 0.081), SRMR = 0.059); and appearance comparison on social media (χ^2^(118) = 274.277, TLI = 0.985, CFI = 0.987, RMSEA = 0.033 (90%CI: 0.028, 0.038), SRMR = 0.078), except for a couple of fit indices that slightly exceeded the cut-off criteria for the body dissatisfaction and appearance comparison scales (see the results in Appendix). Nonetheless, the overall satisfactory fit indices led us to conclude that the measurement invariance was supported for these scales.

However, the invariance testing results were not adequate for the covariate variable of the depressive mood scale, primarily due to a large SRMR value for both the longitudinal (scalar) (χ^2^(56) = 467.645, TLI = 0.953, CFI = 0.945, RMSEA = 0.054 (90%CI: 0.050, 0.059), SRMR = 0.251) and sex (metric) (χ^2^(57) = 614.455, TLI = 0.926, CFI = 0.936, RMSEA = 0.063 (90%CI: 0.058, 0.067), SRMR = 0.252) models. Thus, a sensitivity check analysis was conducted to exclude its potential influence on the results (see Sensitivity Analysis).

### RI-CLPM

The RI-CLPM fit the data well, χ2(1100) = 3648.427, TLI = 0.951, CFI = 0.956, RMSEA = 0.030 (90% CI: 0.029, 0.032), SRMR = 0.032.

The random intercepts showed significant variance, indicating between-person variability in appearance activity on social media (*B* = 0.31, *p* < 0.001); social media-ideal internalization (*B* = 0.34, *p* < 0.001); appearance comparison on social media (*B* = 0.43, *p* < 0.001); and body dissatisfaction (*B* = 0.37, *p* < 0.001) over time. Furthermore, significant correlations were found between the random intercepts (i.e., between-person associations). Over time, adolescents who, on average, more often engaged with appearance activity on social media also reported higher ideal internalization (*r* = 0.47, *p* < 0.001) and appearance comparison (*r* = 0.63, *p* < 0.001) from social media. Surprisingly, more frequent engagement with appearance activity on social media did not correlate with more body dissatisfaction (*r* = −0.02, *p* = 0.712). Nonetheless, higher body dissatisfaction correlated with higher social media-ideal internalization (*r* = 0.61, *p* < 0.001) and appearance comparison on social media (*r* = 0.35, *p* < 0.001), similar to how internalization correlated positively with appearance comparison (*r* = 0.76, *p* < 0.001). Thus, adolescents who, on average, more frequently observed their social media friends posting, sharing, liking, or commenting on appearance content and who engage with these activities themselves also showed strong tendency for internalizing and comparing with social media ideals over time. However, these adolescents did not indicate feeling more dissatisfied with their bodies.

Although the between-person results pointed to associations between appearance activity, internalization, appearance comparison on social media, and body dissatisfaction over time, these were not uncovered at the within-person level (see Table 2). Social media-ideal internalization showed significant auto-regressive paths from W1 to W2 (β = 0.24, *p* = 0.005) and from W2 to W3 (β = 0.37, *p* < 0.001), indicating that adolescents who fluctuated towards higher social media-ideal internalization from their average expected scores in the first wave also fluctuated towards higher internalization in the subsequent waves. Other auto-regressive paths were not significant, meaning that adolescents’ fluctuations towards appearance activity on social media, appearance comparison on social media, and body dissatisfaction did not depend on these fluctuations in the previous waves. Concerning the cross-lagged associations, a fluctuation towards higher social media-ideal internalization in W2 predicted this fluctuation to frequent appearance activity on social media in W3 (β = 0.22, *p* = 0.042) and appearance comparison (β = 0.23, *p* = 0.015). Yet, these effects were not found consistently across all waves. The remaining paths were not statistically significant.

**Table 2:** Within-person associations between appearance activity on social media, social media-ideal internalization, appearance comparison, and body dissatisfaction

	W1 → W2			W2 → W3		
	*B*	β	*p*	*B*	β	*P*
Body dissatisfaction						
Appearance activity	0.01	0.01	0.883	0.03	0.04	0.586
Internalization	0.11	0.13	0.072	0.15	0.18	0.066
Comparison	−0.05	−0.06	0.414	−0.01	−0.02	0.853
Body dissatisfaction	−0.02	−0.02	0.859	0.12	0.11	0.179
Internalization						
Appearance activity	0.04	0.04	0.429	−0.07	−0.06	0.290
Internalization	**0.24**	**0.24**	**0.005**	**0.35**	**0.37**	**<0.001**
Comparison	−0.02	−0.02	0.735	−0.012	−0.01	0.870
Body dissatisfaction	−0.002	−0.002	0.982	0.05	0.04	0.527
Comparison						
Appearance activity	0.07	0.07	0.167	−0.06	−0.06	0.395
Internalization	0.05	0.05	0.484	**0.22**	**0.23**	**0.015**
Comparison	0.05	0.05	0.484	0.06	0.06	0.514
Body dissatisfaction	−0.023	−0.02	0.813	0.06	0.05	0.501
Appearance activity						
Appearance activity	0.07	0.07	0.247	−0.02	−0.02	0.822
Internalization	0.05	0.05	0.500	**0.19**	**0.22**	**0.042**
Comparison	0.01	0.01	0.891	0.03	0.03	0.731
Body dissatisfaction	0.01	0.01	0.899	−0.01	−0.004	0.952

Statistically significant results with *p* < 0.050 are presented in bold

*Appearance activity* Appearance activity on social media, *Internalization* Social media-ideal internalization, *Comparison* Appearance comparison on social media

Next, the mediating roles of social media-ideal internalization and appearance comparison on social media were examined. The model fit was still satisfactory after incorporating the mediating effects with bootstrapped confidence intervals: χ^2^(1100) = 4514.25, TLI = 0.955, CFI = 0.950, RMSEA = 0.035 (90% CI: 0.034, 0.036), SRMR = 0.032. In contrast to expectations, neither internalization (β = 0.01 (−0.01, 0.03), *B* = 0.01) nor appearance comparison (β = −0.001 (−0.02, 0.02), *B* = −0.001) significantly mediated the longitudinal association between appearance activity on social media and body dissatisfaction.

### Differences between Girls and Boys

Differences between girls and boys were examined with the multi-group RI-CLPM. The model with the cross-lagged within-person paths constrained to be equal between girls and boys did not significantly differ from the unconstrained model: χ^2^(24) = 17.877, *p* = 0.809. Both the constrained and unconstrained models fit the data similarly well, χ^2^(2174) = 4975.935, TLI = 0.948, CFI = 0.952, RMSEA = 0.032 (90% CI: 0.031, 0.033), SRMR = 0.038. and χ^2^(2150) = 4955.008, TLI = 0.947, CFI = 0.952, RMSEA = 0.032 (90% CI: 0.031, 0.033), SRMR = 0.037, respectively. This tells us that the within-person associations between appearance activity on social media, social media-ideal internalization, appearance comparison, and body dissatisfaction did not significantly differ for adolescent girls and boys.

### Sensitivity Analysis

To verify the robustness of the results, the associations between appearance activity on social media, social media-ideal internalization, appearance comparison on social media, and body dissatisfaction were examined by running the model while (a) including exposure (i.e., seeing others post, share, comment, or like appearance content on social media) and interactive appearance activities (i.e., one’s own posting, sharing, commenting, or liking of such content) separately, and (b) not incorporating covariates (i.e., BMI, depression, sex, age, time spent on social media). When covariates were excluded, W2 social media-ideal internalization no longer significantly predicted W3 appearance activity on social media (β = 0.20, *B* = 0.19, *p* = 0.057). Additionally, in the model that considered only the exposure to posting, sharing, commenting, and liking of appearance-related social media content (i.e., without active engagement in these activities), increased levels of social media internalization at W2 predicted higher levels of body dissatisfaction at W3 (β = 0.20, *B* = 0.16, *p* = 0.035). Moreover, in the model that focused solely on the active posting, sharing, commenting, and liking of appearance-related content (i.e., without accounting for exposure to others engaging in these activities), adolescents who increased their levels of such activities at W1 reported a higher frequency of appearance comparisons on social media at W2 (β = 0.14, *B* = 0.12, *p* = 0.004). Yet, these associations did not occur consistently across waves.

## Discussion

Engaging with appearance activity on social media has been linked to heightened body dissatisfaction among adolescents (Saiphoo & Vahedi, 2019). Whereas understanding the interplay of the mechanisms underlying this relationship becomes crucial for effective prevention, there is a scarcity of research that provides this insight. The present study examined whether the internalization of social media ideals and appearance comparison mediate the longitudinal association between appearance activity on social media and body dissatisfaction in adolescents. While appearance activity was associated with increased internalization and comparison at the between-person level, no stable associations emerged at the within-person level. These findings do not fully align with the Tripartite Influence Model of body image (Thompson et al., 1999), highlighting distinct patterns in the association between appearance activity on social media and body dissatisfaction in adolescents from the within-person and the between-person perspectives.

### Between-Person Relationships

First of all, the overall rates of body dissatisfaction were relatively low. This alone could partly explain why the current study did not uncover significant within-person relationships over time. As adolescents in this sample reported being rather satisfied with their bodies, there may have been limited room for appearance activity, internalization, and appearance comparison to influence body dissatisfaction and vice versa. With that said, appearance activity on social media was not associated with body dissatisfaction at either the between-person or within-person level.

However, in terms of between-person correlations, across waves, more frequent appearance activity was associated with higher appearance comparison and the internalization of social media ideals. Further, higher internalization and appearance comparison was connected to higher body dissatisfaction. As mentioned earlier, appearance activity did not correlate with adolescents’ body dissatisfaction. These findings correspond with previous results that have indicated that adolescents who engage more with appearance content on social media incline to beauty ideals that such content endorses (Wang et al., 2019). Simultaneously, the higher internalization of idealized appearances and comparison with them is often accompanied by elevated body dissatisfaction (Fardouly & Vartanian, 2015), which corresponds with the current between-person results.

### Within-Person Relationships

Contrary to the hypotheses guided by the Tripartite Influence Model (Thompson et al., 1999), at the level of within-person associations, increased appearance activity on social media, as compared to the usual level of appearance activity across waves, did not relate to such increases in social media-ideal internalization, appearance comparison, and body dissatisfaction in adolescents six months later. The only statistically significant results were that adolescents who more intensely internalized social media ideals subsequently reported elevated appearance comparison and appearance activity on social media. However, these associations occurred only from W2 to W3, limiting their interpretation as substantial and consistent. A sensitivity analysis further showed these associations as non-stable, especially the link from W2 social media-ideal internalization to W3 appearance activity. Importantly, the mediation associations from appearance activity on body dissatisfaction through the internalization of and comparison with social media ideals were not significant.

Overall, the present findings do not align with the expectations drawn from the Tripartite Influence Model (Thompson et al., 1999), because the internalization and appearance comparison with social media ideals did not mediate the relationship between appearance activity on social media and heightened body dissatisfaction over time. More precisely, at a within-person level, appearance activity did not relate to increased body dissatisfaction at all. These results also diverge from the conclusions of cross-sectional studies that pointed to the media-ideal internalization and appearance comparison as mediators for the association between appearance activity on social media and body dissatisfaction in adolescents (Scully et al., 2023; Wang et al., 2019). Using three-wave longitudinal data from Australian adolescents aged 11–16, thin-ideal internalization and social media comparisons mediated the association of the use of visual social media platforms (i.e., Instagram, Snapchat) and engagement in photo-based activities on decreased body satisfaction among adolescents six months later, supporting the Tripartite Influence Model in the context of appearance-centered social media use (Jarman et al., 2024). One of the reasons for the different results can be that Jarman et al. (2024) employed the cross-lagged panel model (CLPM) without the random intercepts, which fails to distinguish between-person and within-person associations over time. Their results likely reflect stable differences between individuals rather than the within-person fluctuations captured in the present study. The CLPM has been criticized for conflating these two distinct types of effects (Hamaker et al., 2015). Indeed, the current results revealed that appearance activity, internalization, appearance comparison, and body dissatisfaction are related differently at the between- and within-person levels, with associations found at the between-person level but not at the within-person level. Given these nuances within the social media effects, further research of this type is needed to elucidate the Tripartite Influence Model’s (Thompson et al., 1999) processes in the relationship between appearance activity on social media and adolescents’ body dissatisfaction.

Nonetheless, these results are in line with other longitudinal research (employing the RI-CLPM as this study did), showing that within-person changes in interactions with appearance content may not consistently relate to later body dissatisfaction in adolescents (Schreurs & Vandenbosch, 2022). It has been suggested that viewing, liking, and commenting on appearance-related content have become so habitual for adolescents that they may already be desensitized to its effects (Schreurs & Vandenbosch, 2022). In addition, the impact on body dissatisfaction may become evident when key variables, such as self-objectification, media-ideal internalization, and appearance comparison, are taken into account (Schreurs & Vandenbosch, 2022). While the desensitization effect might explain these and prior null results, this study did not support the role of the key process variables, particularly the internalization and comparison with social media ideals because neither increased subsequent body dissatisfaction.

Another idea is that appearance activity on social media might only affect adolescents’ body dissatisfaction when it involves relevant friends and peers. The study did not distinguish between interactions with appearance content posted by close friends versus more distant users. Since peer influence on appearance-oriented attitudes is particularly significant during adolescence (Choukas-Bradley et al., 2022), seeing friends promote idealized images may have a greater impact on body image than seeing similar content from unknown individuals. Relatedly, information about the gender (mis)match between the users posting the idealized images and the adolescents engaging with these images might provide valuable insight. Though evidence on this topic remains limited, social comparison theory implies that adolescents might be more influenced by the images that show similar targets, like sharing their gender (Gerber et al., 2018). In follow-up research, it is important to differentiate who posted the content and what specifically adolescents engage with, given the increasingly diverse appearance-centered social media landscape. Asking adolescents about their engagement over the preceding few months, as the current study did, may not capture these details accurately. Experience sampling and data donation methods could be particularly useful for this purpose.

With that said, the null results should also be interpreted in light of the attrition rates. Attrition was relatively high in this study, with fewer than half of the initial sample of adolescents participating in the final wave. The attrition analysis further showed that the drop-out from the second to the third waves was influenced by the frequency of appearance activity and appearance comparison. Adolescents who were more engaged with appearance activity and less inclined toward appearance comparisons were more likely to drop out of the study. The results should be interpreted with caution, considering the potential bias introduced by the attrition and the specific characteristics of the remaining sample.

In addition, this study did not discover that body dissatisfaction, social media-ideal internalization, and appearance comparison would consistently drive engagement with appearance activity on social media. Social media-ideal internalization led to increased appearance comparisons and appearance activity on social media only from W2 to W3. However, it is important to remember the results of the attrition analysis, which showed the attrition rates to be non-random from the second to the third wave of data collection, limiting the generalizability. A sensitivity analysis further pointed to the instability of the association between W2 social media-ideal internalization and W3 appearance activity. Bearing in mind the inconsistency of these associations, such results potentially hint at the possible bidirectional interplay between appearance activity on social media and body image, as well as at a sequential, rather than solely parallel role, of the Tripartite Influence Model’s (Thompson et al., 1999) mediating processes (Rodgers, 2016). While these findings align with some prior research (Jarman et al., 2024; Rodgers et al., 2015), due to their inconclusiveness, further investigation is necessary to understand how body dissatisfaction, internalization, and appearance comparison drive appearance activity on social media.

Within the sensitivity analysis, a few differences emerged between appearance activity directed towards “others”, such as witnessing other users on social media posting, liking, and commenting on idealized content, and activity focused on the “self”, such as adolescents’ own posting, liking, and commenting on similar content. When solely considering the others-focused activities, at the within-person level, increased social media-ideal internalization at W2 predicted increased body dissatisfaction in W3, while this relationship did not appear in the main model or when considering self-oriented activity. On the other hand, increased self-oriented activity in W1 was linked with heightened appearance comparison in W2. These results should be taken cautiously, given their purely exploratory character and the lack of consistency across waves. At the same time, they could be viewed within the context of the recently disputed passive-active dichotomy in social media research. While the idea of passive (other-oriented) use leading to negative outcomes and active (self-oriented) use leading to positive outcomes is widespread, recent evidence opposes this view, because the dichotomy is not clear-cut and the specific outcomes depend on contextual and individual circumstances (Valkenburg et al., 2022). The current findings potentially align with this perspective, because, for both “active” and “passive” forms of appearance activity, negative yet distinct outcomes came forward, namely body dissatisfaction and appearance comparison. Notably, the consequences of appearance-focused social media engagement for body image extend beyond this dichotomy, varying based on the type of content (e.g., selfies, favorable comments) and the form of engagement (e.g., editing, posting) (Vandenbosch et al., 2022). Once again, such a reflection is hindered by the aforementioned limitations.

One aspect to consider is the conceptualization of appearance comparison and the internalization of ideals from social media. The questionnaire did not specifically ask about internalizing thin and muscular ideals, aiming instead to capture a broader spectrum of ideals, thin, muscular, or fit, that adolescents find attractive and that might exacerbate body dissatisfaction. However, it is possible that the internalization and comparison with more realistic body ideals that adolescents find attractive, yet more attainable, was captured as well, like facial ideals (e.g., perfect skin, full lips), that likely do not influence body shape dissatisfaction. Additionally, this study did not assess the direction of the comparisons, whether they were upward, likely reinforcing body dissatisfaction, or downward, potentially alleviating body dissatisfaction. This could partially explain why the association between appearance activity, internalization, comparison, and body dissatisfaction was not observed over time. As literature on social comparison suggests (Knobloch-Westerwick, 2015), interactions with idealized bodies might even promote body satisfaction in cases where self-improvement motives are present, as individuals may view beauty ideals as motivational.

On the methodology side, one explanation of the null results may lie in measuring appearance activity, internalization, appearance comparison on social media, and body dissatisfaction within the time intervals not sufficiently sensitive to capture changes in these variables. This reflection has already appeared in past media effects literature (Maes & Vandenbosch, 2023; Schreurs et al., 2024), proposing that the challenge of detecting media effects may be caused by the fact that these effects arise over shorter (or longer) timeframes than those selected in studies. Intensified tendencies for the internalization of social media ideals and appearance comparison, and the subsequent body dissatisfaction, might emerge as the more immediate consequences of appearance activity on social media than after a six-month period. In that case, longitudinal designs with shorter time frames (e.g., experience sampling studies) could be a fruitful direction in this research area. That said, long-term longitudinal studies that span several years also present a valuable avenue. The present research examined appearance-related social media use and body image over a one-year period, which captures a relatively brief period of adolescent development. To gain a more comprehensive understanding of this relationship, future research could benefit from combining experience sampling studies and focusing on short-term effects, with long-term studies that span the full adolescence period.

Relatedly, a reflection on the importance of sensitivity to calendar moments has recently emerged in digital media effects literature (Vandenbosch et al., 2025). The present study collected data in June (W1), December (W2), and the following June (W3), with significant within-person effects occurring only between W2 and W3. Regarding the topic of body image, adolescents may be more susceptible to the effects of digital media during the summer months due to wearing typically more revealing clothing (Vandenbosch et al., 2025). This aspect could have underlaid the observed inconsistent findings across waves and should be considered more thoroughly in follow-up research.

### Differences Between Girls and Boys

Despite girls being more invested in appearance social media interactions (Paddock & Bell, 2021) and because they are to a higher extent socialized to accomplish attractive ideals (Choukas-Bradley et al., 2022), the within-person associations between appearance activity, internalization, appearance comparison on social media, and body dissatisfaction did not differ between girls and boys. This is consistent with previous research, which showed that the connection between the time spent on appearance-centered social media and lower body satisfaction, as mediated by social and appearance comparison and thin-ideal internalization, was the same for adolescent girls and boys aged 11–17 (Jarman et al., 2021b, 2024). Similarly, time spent on social media and appearance-focused activities related to negative body image across studies, regardless of gender (Saiphoo & Vahedi, 2019). It should be noted that, while the present study assessed binary categories with which the adolescents identified, variations in the terminology of “sex” and “gender” across studies may somewhat limit comparability. An increasing number of studies highlight that adolescents with diverse gender identities have unique body image experiences (McGuire et al., 2016). These distinct experiences may also shape how they engage with appearance activity on social media and the subsequent impact on body dissatisfaction. For instance, gender-diverse adolescents might interact with different appearance ideals, as androgynous body ideal, combining both masculine and feminine traits, is particularly prominent among non-binary and transgender people (Galupo et al., 2021). Future research is encouraged to look beyond the gender binary and include adolescents with diverse gender and sexual identities, as these groups are often overlooked, as well as into the person-specific effects of individual adolescents (Valkenburg et al., 2024). The need to account for individual moderators, such as different motivations for internet use (e.g., seeking appearance feedback, social support) has already been emphasized (Rodgers, 2016). Within the context of these findings, and in line with the uses and gratification perspective, the appearance activity possibly reinforces body dissatisfaction for some adolescents while having no impact, or even promoting body satisfaction for others, that is dependent on individual experiences and motivations.

### Limitations and Future Directions

This study followed the Tripartite Influence Model of body image (Thompson et al., 1999), employing a longitudinal study design and a robust sample of adolescents. However, further research may consider looking at multiple aspects of adolescent body image and other essential processes that link it to appearance-related social media use. Adolescent body image encompasses not only body (dis)satisfaction but also positive body image characteristics, such as body appreciation and resilience to appearance ideals and negative appearance feedback (Maes et al., 2021). With that said, positive body image can serve as a crucial protective factor for well-being, extending beyond body dissatisfaction (Tylka & Wood-Barcalow, 2015). To fully understand the impact of appearance-related activities on social media on adolescent body image, it is essential to include positive body image characteristics in future research. Regarding sampling bias, attrition from W2 to W3 was related to the reported frequency of appearance activity and appearance comparison. This limitation should be considered when interpreting the results.

Although age was included as a covariate, the present study did not delve into specific developmental factors that might influence the relationships between appearance activity and body dissatisfaction. In particular, pubertal status may play an important role, because early- or late-maturing adolescents can experience varying levels of body dissatisfaction. For example, early maturation appears to increase body dissatisfaction in girls but reduce it in boys, due to different body ideals (Ricciardelli & Yager, 2016). Consequently, pubertal timing could shape the responses to appearance-focused content on social media, because it motivates adolescents to different social media behaviors (Swirsky et al., 2022). Exploring the different developmental trajectories of engagement with appearance activity and body dissatisfaction, as influenced by pubertal status, could provide valuable insights in future research.

Other limitations pertain to the context of data collection. First, the data were collected via online questionnaires distributed in an agency’s online panel. This approach provided limited control over the questionnaire completion process, because adolescents’ parents received the questionnaire link via email and adolescents could fill it out at anytime and anywhere. Consequently, some adolescents may have paid limited attention to the questionnaire or completed it under the influence of peers or their parents. Furthermore, parents acted as gatekeepers, who could have excluded their offspring from the study after reviewing the questionnaire. This may have led to the underrepresentation of certain groups of adolescents, such as those with past negative experiences with social media or body image concerns, limiting the generalizability of the findings. Second, the data were collected during the COVID-19 pandemic, introducing unique circumstances that affected adolescents’ social media use and other areas of their lives. In Czechia, the months leading up to the first wave of data collection in June 2021 were marked by a ban of offline schooling (Slabá, 2022). Overall, there were accounts of increased social media use among adolescents during the pandemic (Marciano et al., 2022). At the same time, the measures implemented, such as physical distancing, posed significant challenges to youth’s mental health (Meherali et al., 2021). While body dissatisfaction rates were relatively low in the current study sample, these findings should be considered in light of the pandemic circumstances, and further replication research with other samples is needed.

Another limitation relates to the way that body dissatisfaction was measured. The scale was not administered in its entirety, as items that assessed the same body parts in a reverse manner were omitted. The scale has also been validated for adolescents aged 13 and older (Garner, 2004). Given that the present study also included early adolescents as young as 11, it is important to acknowledge potential limitations in measuring body dissatisfaction within this age group. For instance, young adolescents who have not yet experienced pubertal changes in body shape and weight may not interpret the scale items in the same way, potentially limiting the accuracy of body dissatisfaction measurement. Furthermore, the measurement did not differentiate between specific aspects, such as thinness, body weight, body fat, or muscularity. Recently, concerns have been raised about the inadequate measurement of body image and disordered eating in adolescent boys with instruments that are not specifically designed to capture key aspects of boys’ body image (Hansson & Schmidt, 2025). For boys in particular, muscularity and body fat are significant components of their body image (Tylka, 2011). These aspects were not assessed and, as a result, some crucial components of adolescent boys’ body dissatisfaction may have gone undetected, potentially diminishing the connection with appearance activity. A more complex approach to adolescents’ body dissatisfaction with a particular focus on the adequate content validity of the utilized scales is essential in future research. Furthermore, a greater nuance in the conceptualization and measurement of appearance activity, appearance comparison, and internalization variables could be valuable for future research. For instance, distinguishing between upward and downward comparison processes, as well as the exploration of the internalization of diverse body types, such as thin versus fit ideals, might show different links between appearance activity and body dissatisfaction. Adolescents can also engage in appearance activity with different user targets, such as friends versus more distant users, and interact with different kinds of content that serves different purposes, like fitspiration to promote fitness and lean ideals versus body positivity to encourage body acceptance. Clarifying these nuances is therefore an important task for follow-up research.

## Conclusion

As a consequence of intensive body image development, adolescents frequently engage in appearance activity on social media to learn about peer norms for physical appearance and receive feedback on their looks. While such activity can negatively impact body dissatisfaction in adolescents, there is a lack of research enlightening the particular mechanisms that underlie this relationship. Gaining insight into how appearance activity contributes to body dissatisfaction is key for advancing research in this area and informing prevention efforts aimed at reducing body dissatisfaction in adolescents. The present study examined whether the internalization of social media ideals and appearance comparisons mediate the relationship between appearance activity on social media and body dissatisfaction over time. Contrary to the predictions of the Tripartite Influence Model of body image, the findings did not find support for these mediation hypotheses. At the between-person level, higher average levels of appearance activity were associated with the increased internalization of social media ideals and more frequent appearance comparisons over time. However, these between-person findings did not align with the within-person results. Individual fluctuations in appearance activity were not consistently linked to corresponding changes in appearance comparison, the internalization of social media ideals, or body dissatisfaction in subsequent waves. At the within-person level, the heightened internalization of social media ideals predicted more frequent appearance activity and appearance comparisons in a later wave, though not consistently across all waves. This study shows that, while greater engagement in appearance activity on social media is associated with a heightened inclination toward social media ideals, it does not inevitably intensify later body dissatisfaction in adolescents. These findings somewhat challenge the widespread perception that the social media effects on adolescent body image are universally detrimental, although it is important to consider the limitations of the sample, including higher attrition rates and data collection during COVID-19 regulations. Nonetheless, as the results outline, there does not yet appear to be sufficient evidence to promote targeting internalization and comparison with social media ideals as effective strategies for reducing later body dissatisfaction in adolescents. The present study contributes to the understanding of appearance-focused social media use and body dissatisfaction in adolescence by highlighting the distinct patterns observed at the within-person and between-person levels, offering insights for future work on this crucial aspect of adolescent body image.

## Appendix

Tables 3, 4

**Table 3:** Results of longitudinal invariance

Construct	Invariance type	X2 (df)	TLI	CFI	RMSEA	SRMR
Body dissatisfaction	Configural	306.357 (75)	0.974	0.981	0.035 (0.031, 0.039)	0.083
	Metric	327.666 (83)	0.975	0.980	0.034 (0.031, 0.038)	0.081
	Scalar	370.596 (93)	0.975	0.978	0.035 (0.031, 0.038)	0.078
Internalization	Configural	283.991 (72)	0.980	0.986	0.034 (0.030, 0.039)	0.025
	Metric	307.996 (80)	0.981	0.985	0.034 (0.030, 0.038)	0.029
	Scalar	334.565 (90)	0.981	0.984	0.033 (0.029, 0.037)	0.031
Comparison	Configural	62.506 (42)	0.997	0.998	0.014 (0.006, 0.021)	0.029
	Metric	70.603 (48)	0.997	0.998	0.014 (0.006, 0.020)	0.032
	Scalar	83.270 (56)	0.997	0.998	0.014 (0.007, 0.020)	0.036
Depressive mood	Configural	409.886 (42)	0.934	0.958	0.059 (0.054, 0.064)	0.254
	Metric	614.455 (57)	0.926	0.936	0.063 (0.058, 0.067)	0.252
	Scalar	467.645 (56)	0.953	0.945	0.054 (0.050, 0.059)	0.251

*Internalization* Social media-ideal internalization, *Comparison* Appearance comparison on social media

**Table 4:** Results of invariance between girls and boys

Construct	Invariance type	X2 (df)	TLI	CFI	RMSEA	SRMR
Body dissatisfaction	Configural	462.447 (184)	0.975	0.978	0.035 (0.031, 0.039)	0.032
	Metric	490.875 (189)	0.974	0.976	0.036 (0.032, 0.040)	0.069
	Scalar	580.948 (194)	0.967	0.970	0.040 (0.036, 0.044)	0.060
Internalization	Configural	1514.312 (184)	0.913	0.901	0.076 (0.073, 0.080)	0.041
	Metric	1532.212 (189)	0.903	0.913	0.075 (0.072, 0.079)	0.043
	Scalar	1589.565 (189)	0.899	0.909	0.077 (0.074, 0.081)	0.059
Comparison	Configural	116.374 (110)	0.999	0.999	0.007 (0.000, 0.016)	0.033
	Metric	146.114 (114)	0.997	0.997	0.015 (0.006, 0.022)	0.064
	Scalar	274.277 (118)	0.985	0.987	0.033 (0.028, 0.038)	0.078
Depressive mood	Configural	200.408 (110)	0.987	0.989	0.026 (0.020, 0.031)	0.041
	Metric	614.455 (57)	0.926	0.936	0.063 (0.058, 0.067)	0.252
	Scalar	320.610 (118)	0.974	0.976	0.037 (0.032, 0.042)	0.061

*Internalization* Social media-ideal internalization, *Comparison* Appearance comparison on social media
